# A National Department of Health

**Published:** 1906-12

**Authors:** J. Pease Norton

**Affiliations:** Yale University


### A National Department of Health.

**Professor J. Pease Norton, of Yale University, Department of Social Science, Advocates the Establishment of a Department of Health at Washington as an Administrative Branch of the Government.**

EDITOR BUFFALO MEDICAL JOURNAL:

SIR:—Pasteur wrote: It is within the power of man to rid himself of every parasitic disease.

The time has now arrived, in the judgment of many persons, for establishing a National Department of Health at Washington to wage warfare against the preventable diseases of mankind. In a similar way, the United States Department of Agriculture has expended during the last ten years fifty millions of dollars in a splendid fight against the diseases of plants and animals.

The fearful wastes of death and sickness and the dreadful havoc wrought can never be described. The fiercest battles ever fought left no such bloody trails, even when the mailed hand of war smote cruelest and harshest, as the crimson boulevards—*ten-death-chariots-wide*—traced by the passing finger touches of pneumonia or consumption wastes within a week's end span. Along these ghastly boulevards will be strewn, before twelve months are gone, more blasted hopes and broken hearts than all the countless grinning skulls slain in fair fights and whitening battlefields since time began.

Could the 750,000 persons in the United States marked for death during the next twelve months from preventable causes voice in a threniad verdict their conviction, in a last solemn *moriturii salutamus,* who can doubt what would be their admonition: that good health is more precious than rubies, and a long life well lived fairer than beaten gold. Who can doubt, if these measures were before the nation, how they would cast their verdicts? As the slaves chained to the chairs of the conquerors in the triumphs that are gone, so the passing hours silently remind us: "And we too are mortal."

In the accompanying paper read before the American Association for the Advancement of Science, partial data have been assembled concerning the magnitude of these wastes and the possibilities of prevention. The great need is for the awakening of the nation to the splendid remedies which the great medical and sanitary experts could devise, if properly organised for the task.

Could only the busy editors of the nation, who guide all movements of humanity and progress, co-operate in bringing this fearful destruction vividly before their readers, no greater good could be accomplished; and in the years to come, many a man now marked for an early death, when celebrating his ninetieth birthday among his children, would thank his stars for a progressive press.

With best wishes and thanking you in advance for whatever co-operation may be rendered in this agitation for inaugurating a National Department of Health, which must go on until the purposes are allowed and the means provided, believe me

J. PEASE NORTON.

Yale University,
November 1, 1906.

---

IF in a case of suspected appendicitis the point of greatest tenderness is situated at a higher level than customary, it is important after opening the abdomen to always examine the gallbladder for the presence of gallstones.—*International Journal of Surgery.*

